# Medical Association of Central New York

**Published:** 1899-12

**Authors:** C. A. Van Der Beek


### Society Proceedings.

### MEDICAL ASSOCIATION OF CENTRAL NEW YORK.

Reported BY C. A. VAN DER BEEK, M. D., Secretary.

THE thirty-second annual meeting of the Medical Association of Central New York was held at the Yates Hotel, Syracuse, October 17, 1899.

The meeting was called to order at 10.55 a. m., by the president, Dr. E. L. MOONEY, of Syracuse.

THE PRESIDENT. —The first order of business is the reading of the minutes of the previous meeting.

The secretary, Dr. VAN DER BEEK.—Inasmuch as a stenographic report of the minutes of the last meeting was published in the transactions and a copy sent to every member in attendance, I would move that the reading of the minutes be dispensed with. Seconded and carried.

The president appointed the following committees: *Business Committee.*—F. W. Sears, Onondaga; Wm. A. Howe, Ontario; A. A. Young, Wayne.

*Committee on Credentials.*—H. L. Chase, Wayne; E. W. Ruggles, Monroe; Frederick Preiss, Erie.

*Reception Committee.*—D. M. Totman, E. J. Wynkoop, H. B. Hawley, Onondaga;

*Committee on Ethics.*—Lucien Howe, Erie; J. P. Creveling, Cayuga; Nathan Jacobson, Onondaga.

*Publication Committee.*—Wm. C. Krauss, Erie; S. L. Elsner, Monroe; C. A. Van der Beek, Monroe.

Dr. JOHN GERIN, of Auburn, first-vice president, was called to the chair while the president delivered the annual address on The attitude of the general practitioner toward the specialist.

Dr. SEARS, Onondaga.—Mr. vice-president, I move a vote of thanks to the president for his address. Carried.

The following members and delegates registered :

*Broome.*

Seymour, R. A.

*Cayuga.*

| | | |
|---|---|---|
| Austin, L. E. | Gerin, John | Hall, A. L. |
| | Laird, W. R. | |

*Chenango.*

Phelps, R. H.

*Cortland.*

| | | |
|---|---|---|
| Andrews, L. C. | Foosher, F. H. | Higgins, J. W. |

*Erie.*

| | | |
|---|---|---|
| Benedict, A. L. | Fronczak, F. E. | Howe, Lucien. |
| | Krauss, Wm. C. | |

*Fulton.*

Bacon, C. Y.

*Genesee.*

Wm. D. Johnson.

*Madison.*

| | | |
|---|---|---|
| Bailey, E. R. | Goff, I. N. | Miller, E. S. |

*Monroe.*

| | | |
|---|---|---|
| Angell, E. B. | Greene, George W. | Ozman, I. D. |
| Ballentine, E. N. | Greenleaf, C, A. | Pennington, A. C. |
| Boswell, C. | Hemus, A. A. | Potter, M. C. |
| Berkman, J. S. | Ingersoll, J. | Ruggles, Elwood |
| Brown, A. H. | Jones, W. B. | Sullivan, J. H. |
| Brown, W. M. | Leary, M. E. | Thomas, S. W. |
| Comfort, C. B. | Murphy, P. | Van der Beek, C. A. |
| Cochane, William B. | Boddy, Edmund C. | Weaver, J. E. |
| Conklin, Wm. S. | Mooney, Thomas T. | White, George F. |
| Elsner, S. L. | Nugent, E. G. | Wallace, W. T. |

*Ontario.*

| | | |
|---|---|---|
| Allen, D. S. | Baright G. E. | Beahan, A. L. |
| Howe, Wm. A. | Merritt, C. P. | Spalding, F. W. |

*Orleans.*

Starer. F. B.

*Onondaga.*

| | | |
|---|---|---|
| Baum, Henry C. | Johnson, J. R. | Rutland, A. S. |
| Brood G. B. | Jacobson, N. | Rood, A. B. |
| Belknap, E. W. | Kellogg, W. C. | Randall, A. B. |
| Babcock, A. B. | Leary, J. H. | Russell, W. G. |
| Blumenthal, C. A. | Loveland, B. C. | Snow, S. F. |
| Cullings, M. H. | Lewis, G. G. | Stephenson, F. H. |
| Craton, S. B. | Miller, A. B. | Sears, J. W. |
| Didama, H. D. | Mooney, E. L. | Slocum, F. W. |
| Elsner, H. L. | Mercer, A. C. | Totman, D. M. |
| Easton, F. E. | Marlow, W. | Vadeboncoeur, A. F. |
| Flaherty, J. H. | McClary, C. E. | Van de Warker, Ely |
| Felton, C. A. | Munson, W. W. | Vedder, A. M. |
| McLennan, R. C. | Mercer, A. | Wright, H. B. |
| Halsted, T. H. | McMorrow, F. | Wiggins, H. |
| Hawley, H. B. | Pearson, R. H. | Wynkoop, E. J. |
| Heffron, J. L. | Price, G. M. | |

*Oswego.*

| | | |
|---|---|---|
| Cooley, R. N. | Crockett, R. S. | Doane, H. M. |
| Dowd, P. M. | Rainier, E. | Todd, William C. |
| Wells, W. M. | Wilcox, H. P. | |

*Schuyler.*

Bernett, M. S.

*Seneca.*

| | | |
|---|---|---|
| Everets, D. F. | Madden, J. E. | Onan, R. E. |
| Purdy, J. S. | Russell, William. | |

*Syracuse.*

Ayling, W. J.

*Wayne.*

| | | |
|---|---|---|
| Arnold, J. N. | Brownell, M. A. | Carmer, M. E. |
| Carr, R. S. | Lapp, E. H. | Myers, J. F. |
| Smith, L. H. | Young, A. A. | |

*Wyoming.*

Palmer, George W.

*Yates.*

Doubleday, C. E.

THE PRESIDENT.—The program is now in the hands of the business committee.

DR. SEARS.—Before we proceed with the addresses, I would like to ask permission to invite Dr. Isabel McMillen, of Oswego, to take part in the exercises of the day, if there is no objection. It was so ordered.

Papers were then read as follows :

Nervous dyspepsia, by B. C. Loveland, Syracuse.

A plea for the more frequent use of mydriatics in so-called ophthalmias, by Lucien Howe, Buffalo.

On the resuscitation of the newborn by Laborde's method, by Francis E. Fronczak, Buffalo.

Treatment of epilepsy in its incipient stages, by Wm. P. Spratling, Sonyea. Report of two cases of sub-acute myelitis, by Edward B. Angell, Rochester.

THE PRESIDENT.—I wish to invite the members of the Central New York Medical Association to go to the Medical College, immediately after luncheon, where Dr. Clark, the professor of physiology, has kindly consented to show the circulation in the beef-heart in active operation; it will be a very interesting sight, no doubt. The afternoon session will begin promptly at 2 o'clock.

All delegates who are eligible to permanent membership will please come to the secretary and enroll their names immediately at the beginning of the afternoon session.

Afternoon session convened at 2.30 p. m.

THE PRESIDENT.—There is one case which is not down on the program. Dr. Stebbins, of Onondaga, has a patient in the side-room which he will tell you about now and which those of you who care to, can step out and examine.

DR. STEBBINS.—The patient which I have here in the ante-room has suffered from cataract. The young man is 33 years of age and he has been operated upon for a cataract. His complications are a detachment of the retina and nystagmus. The young man came to me last March and had a cataract, which I removed. I did not perform the modified linear extraction, but removed the lens entire. The patient had a great deal of pain, but no redness or inflammation, and he is now in the ante-room and you can see him. You will find a very good looking eye, and he has now, with glasses, 20-70 vision.

The secretary read two letters which had been received by him with the request that they be read at the meeting of this Association, one dated Detroit, October 7, 1899, and the other Savannah, Georgia, June 6, 1899.

Dr. GERIN.—I move that we adopt the sentiment or resolution, if you please, as it is embodied there, and that we instruct our secretary to communicate with the American Medical Association, setting forth our urgent request for the promotion of the Surgeon-General to the rank of Major-General. I think myself that is the proper place for him to be. He should be where he won't have to take orders from every boy that ranks him, because he has the star of Brigadier-General. I think frequently the members of our profession do not obtain the rank in the army or in the navy or in the militia which their service and intelligence and education entitle them to.

Seconded by Dr. Arnold and carried.

Dr. MOONEY.—The first paper which was read with regard to state medical education, what will be done with that? Dr. Krauss, what have you to say about that?

Dr. KRAUSS.—I heard part of it and I read the letter over while seated with the secretary; I think it would be a good plan for the society to adopt the idea mentioned in the paper. I make a motion to that effect, that we subscribe to the statements and place our society in line with the ideas brought forth in that letter.

Seconded by Dr. Gerin and carried.

Treasurer's report, by Dr. S. L. ELSNER.

ROCHESTER, N. Y., October, 1899.

| | | | | |
|---|---|---|---|---|
| 1898. | | | DR. | |
| October | 18. | Cash on hand at last report | $115.02 | |
| " | 18. | Collected dues at Auburn, 1898 | 110.00 | |
| " | 18. | Collected fees " " | 26.00 | |
| December | 1. | Collected from Dr. Woodruff | 1.00 | |
| 1899. | | | | |
| March | 10. | Check returned from printer, A. T. Brown | 7.00 | |
| | | | | $259.02 |

| | | | |
|---|---|---|---|
| 1898. | | | CR. |
| October | 18. | Janitor and help | $ 1.00 |
| " | 18. | Wm. C. Krauss, postage, etc | 5.50 |
| November | 9. | Miss Knapp, stenographer | 24.52 |
| " | 9. | Bills to post express | 20.25 |
| " | 9. | John C. Moore, book-binding | 2.00 |
| " | 9. | Secretary's salary to October, 1898 | 10.00 |

| 1899. | | | Cr. | |
|---|---|---|---|---|
| January | 23. | Exchange on check | $ .15 | |
| " | 23. | Postage and stationery | 1.00 | |
| March | 8. | A. T. Brown, Higgins' plate | 7.00 | |
| July | 4. | A. T. Brown, printing | 2.85 | |
| " | 30. | A. T. Brown, Transactions | 65.00 | |
| Sept. | 9. | C. A. Vander Beek, postage, envelopes, printing, etc | 45.00 | |
| | | Total expenses | | $184.27 |
| | | Cash to balance on hand | | 74.75 |
| | | | | $259.02 |

Dr. Gerin.—I move that the report be accepted. Seconded by Dr. Angell and carried.

The election of officers was the next order of business and resulted as follows: President, John Gerin, Auburn; first vice-president, Dr. Lucien Howe, Buffalo; second vice-president, A. L. Beahan, Canandaigua; secretary, C. A. Van der Beek, Rochester; treasurer, S. L. Elsner, Rochester.

Dr. Reynolds, of the committee on membership, reported that the following physicians had complied with the requirements of the Association and were eligible to membership: S. E. Austin, Cayuga; F. E. Fronczak, Erie; W. D. Johnson, Genesee; E. C. Boddy, W. T. Wallace, C. V. C. Comfort, C. O. Boswell, Monroe; G. B. Broad, F. E. Easton, A. B. Babcock, O. A. Blumenthal, S. B. Craton, T. H. Halsted, Onondaga; F. W. Spaulding, C. P. W. Merritt, Ontario; E. Ranier, Oswego; W. L. Russell, Seneca; M. L. Bennett, Schuyler; R. S. Carr, E. H. Lapp, J. F. Myers, Wayne.

Dr. Angell.—I move the names as read be admitted to membership.

Seconded by Dr. Arnold and carried.

Dr. Sears, of the business committee: Owing to the lateness of the hour, it seems wise to the business committee to limit the papers to ten minutes and the discussions to five, for the reason we have a large number of papers to get through with and the time is limited. It is also requested that each one who reads a paper will leave a copy with the secretary.

Presentation of patient sixteen months after modified Kraske's operation for extirpation of rectum, by S. L. Elsner, Rochester.

Personal experience with fracture of the hip, by G. W. Green, Auburn.

The missile and the weapon, by A. L. Hall, Fair Haven.

Uterine hemorrhage, by A. L. Beahan, Canandaigua.

The clinical thermometer as a germ carrier, by W. L. Conklin, Rochester.

Traumatic nephritis, by J. P. Creveling, Auburn.

A case of carcinoma of the prostate gland, associated with stone in the bladder, by Nathan Jacobson, Syracuse.

Cephalagia and tic douloureux from accessory sinus affections, by Sargent F. Snow, Syracuse.

My experience with formalin, by Ely Van de Warker, Syracuse.

Dr. Gerin.—Last year I gave notice to move an amendment at this meeting, changing Article I. of the Constitution. The article now reads that this organisation shall be called "The Medical Association of Central New York." I gave notice to amend it to read, "The Medical Association of Central and Western New York," or, "Central-Western New York." I believe that has been suggested today as meeting the approval of some. If the Association is prepared for the amendment, I will move its adoption.

Seconded by Dr. Van de Warker.

Dr. Van der Beek.—I would say that at the last meeting the President, Dr. Krauss, made certain recommendations and appointed a committee to consider them.

After some discussion, Dr. L. A. Weigel said: It seems to be the general impression that the committee is still in existence. The committee appointed on the president's address has made its report and I presume its labors are ended. If any amendments are to be acted upon, it will be necessary to appoint a new committee for that purpose. There is no committee now.

Dr. Gerin.—I move that a new committee be appointed.

Seconded by Dr. Van der Beek and carried.

The president appointed as such committee: Dr. Jacobson, as chairman; Dr. Angell, of Rochester, and Dr. Krauss, of Buffalo.

The following papers were then read by titles:

Taking inventory of a nervous case, by Wm. C. Krauss, Buffalo.

Erysipelas, by A. A. Young, Newark.

Cystitis, by C. C. Thayer, Clifton Springs.

Some of the causes and effects of mouth-breathing, by J. M. Ingersoll, of Rochester.

Report of pyloric and cardial tumor, cured by jejunostomy and physiologic rest of stomach, by A. L. Benedict, Buffalo.

The treatment of pulmonary tuberculosis, by Parker Murphy, Rochester.

Rare case; exhibition of specimen and result of operation, by J. Henry Dowd, Buffalo.

Dr. GERIN.—I would like to move a vote of thanks to the retiring president for the very able and courteous manner in which he has presided at this meeting.

Dr. MURPHY.—I would amend that motion by including our very able secretary, who has done so much valuable work. Seconded by Dr. Sears and carried.

Dr. ANGELL.—I move that the Society extend a vote of thanks to the Onondaga Medical Society for the very generous way in which they have entertained the Association today.

Seconded by Dr. Gerin. Carried.

The meeting then adjourned to meet the third Tuesday in October, 1900, at Rochester, N. Y.

